# SARS-CoV-2 Infection in the Second Trimester of Pregnancy: A Case Report of Fetal Intraventricular Hemorrhage After Critical COVID-19 Infection and a Brief Review of the Literature

**DOI:** 10.7759/cureus.48659

**Published:** 2023-11-11

**Authors:** Antonella Vimercati, Rosalba De Nola, Miriam Dellino, Lorenzo Vinci, Ilaria Ricci, Antonio Malvasi, Gianluca Raffaello Damiani, Maria Gaetani, Bruno Lamanna, Ettore Cicinelli, Cecilia Salzillo, Andrea Marzullo, Leonardo Resta, Eliano Cascardi, Gerardo Cazzato

**Affiliations:** 1 Department of Precision and Regenerative Medicine and Ionian Area (DiMePRe-J), University of Bari Aldo Moro, Bari, ITA; 2 Gynecology, University of Bari Aldo Moro, Bari, ITA; 3 Section of Pathology, Department of Emergency and Organ Transplantation, University of Bari Aldo Moro, Bari, ITA; 4 Department of Pathology, University of Bari Aldo Moro, Bari, ITA; 5 Pathology Unit, FPO-IRCCS Candiolo Cancer Institute, University of Turin, Turin, ITA

**Keywords:** ace receptors, de-ciduitis, placental, trimester, pregnancy, sars-cov-2, covid 19, hemorrhage, intraventricular, fetal

## Abstract

More than three and a half years have passed since the start of the coronavirus disease 2019 (COVID-19) pandemic, caused by severe acute respiratory syndrome coronavirus 2 (SARS-CoV-2), and there have been several studies in the literature about the different damage and symptom patterns related to the condition; particular attention has been paid to the transmission of the disease from pregnant mothers to fetus.

In this report, we present the case of a 36-year-old patient with a history of two cesarean sections (CS), two miscarriages, and hypothyroidism on replacement therapy, who contracted COVID-19 during the 15th week of gestation. Ultrasound (US) examination at 22 weeks revealed regular fetal biometry and bilateral ventriculomegaly, highly suggestive of massive intracerebral hemorrhage. The patient opted for the interruption of pregnancy. Given the critical maternal COVID-19 complications, especially tracheoesophageal fistula and the patient's two previous cesareans, we decided on an abortive CS at 23 weeks of gestation, and the samples were sent to the Pathology Department. Histologic analysis showed massive intervillous deposition of fibrin and inflammatory infiltration with hotspots of necrotic deciduitis and confirmed massive cerebral hemorrhage in the fetus. This morphological appearance was consistent with COVID-19 infection and probable fetal oxygenation compromise related to deciduitis. Immunoexpression of anti-SARS-CoV-2 S1 antibody was almost entirely positive at the level of syncytiotrophoblast cells and maternal leukocytes in the absence of a clear signal in the fetal circulation. Conversely, in the brain, immunoexpression of angiotensin-converting enzyme 2 (ACE2) and the S1 subunit of the spike protein of SARS-CoV-2, detected by a monoclonal antibody, was almost entirely negative, suggesting that there was no infection in the brain and that the massive intraventricular hemorrhage was probably a secondary effect of placental damage.

## Introduction

The coronavirus disease 2019 (COVID-19) pandemic, caused by severe acute respiratory syndrome coronavirus 2 (SARS-CoV-2), has had a huge impact on the scientific community worldwide, prompting scientists and researchers to undertake massive efforts to comprehend (especially in the first few months after the pandemic began) the disease mechanisms and to predict what the evolution of the condition entails [1]. In more than three and a half years since the start of the pandemic, much has been written and said about the various damage and symptom patterns related to COVID-19, and the transmission of the disease from pregnant mothers to fetuses has received significant attention and scrutiny [2-4] along with the more varied organ involvements such as, among the less frequent, the skin [5], the gastroenteric system [6], and even the encephalon [7]. Although the underlying mechanism has not yet been fully elucidated, several studies have suggested that the placenta acts as a 'barrier', capable of stemming the spread of virions and preventing potential transmission to the fetus [8-9]. We have already reported, in a previous paper, a case of twin pregnancy where severe alterations in the placental discs were described, but without transmission to the fetus [8]. A renewed scientific interest is now emerging about the sequence of placental COVID-19-related modifications with deciduitis, maternal-fetal malperfusion (MFM), fetal ipossic evolution, fetal immune and proinflammatory activation, severe neurological outcomes, and even congenital defects [1-10].

In this report, we engage in a comprehensive discussion about the maternal clinical aspects, the fetal ultrasound (US) features, and pathological findings related to an uncommon case of critical maternal and fetal-neonatal outcomes related to COVID-19 during the second wave of the pandemic, which was dominated by the Alpha variant of SARS-CoV-2.

## Case presentation

A 36-year-old patient with a history of two cesarean sections, two miscarriages, and hypothyroidism in therapy, contracted COVID-19 during the 15th week of gestation. First-trimester combined screening showed a low risk for chromosomopathies with regular fetal morphology and nuchal translucency in the normal range. The patient had interacted with a SARS-CoV-2-positive person at 14 weeks + one day of pregnancy and, afterward, she had started showing symptoms, such as fever, chills, lower limb weakness, and thoracic oppression.

After 24 hours (one day) of hypoxemic status, the rapid deterioration of her clinical conditions led to step-up hospitalization in the ICU. The patient underwent noninvasive ventilation (NIV) and quickly proceeded to orotracheal intubation (OTI). During hospitalization, the patient was administered enoxaparin sodium (Clexane) 4000 IU as prophylaxis for deep vein thrombosis (DVT) together with cortisone and an antibiotic (fosfomycin). The patient did not have any bacterial infection. After a few days, she underwent a percutaneous tracheostomy, and subsequently a temporary (one-day) synchronized intermittent mandatory ventilation (SIMV). Coagulation test results were within the normal range except for an elevation in D-dimer (3500 microgrammi/ml). Two days later, at 18 weeks + three days of pregnancy, and after 15 days of intubation, the patient woke up, and four days later (19 weeks + 0 days), the COVID-19 test returned negative. The patient was transferred to a medical internal clinic for convalescence; a week later, she experienced a tracheoesophageal fistula as a post-intubation complication and underwent a percutaneous endoscopic gastrostomy (PEG) feeding.

**Figure 1:**
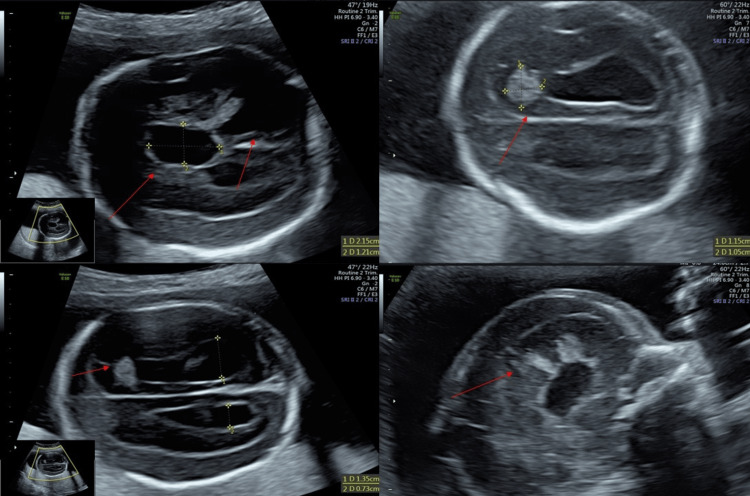
Various scan findings (A) Oblique axial scan: evidence of structure disruption with central pseudocyst (red arrow) (21.5 x 12.5 mm) and massive hypoechogenicity in the posterior horn as a massive hemorrhage (red arrow). (B) Axial scan: a clearly visible hyperechogenic lesion (11.5 x 10.5 mm) in the anterior proximal horn associated with a blood clot (red arrow) and the hyperechogenicity of the ventricular wall due to chemical ventriculitis. The ventriculomegaly is probably related to aqueduct obstruction. (C) Oblique axial scan: evidence of bilateral ventricular hemorrhage with a blood clot in the anterior horn (red arrow). (D) Midsagittal scan: evident hypoechogenic pseudocyst and some hyperechogenic lesions (red arrow)

During hospitalization, the patient underwent morphological US at 21 weeks and five days; fetal biometry results were compatible with the period of gestation, and the fetus showed bilateral hydrocephalus with third ventricle dilatation, which required the involvement of our High-Risk Pregnancy Centre. The patient underwent a US examination (GE Voluson E10, Kretz, US machine); fetal biometry was shown for 22 weeks and bilateral ventriculomegaly was highlighted, with posterior horn measuring 19 mm and anterior horn measuring 8 mm. An oblique axial scan revealed disruption of cerebral tissue with central pseudocyst (21.5 x 12.5 mm) and massive hypoechogenicity in the posterior horn as a massive hemorrhage (Figure 1A); a suprathalamic axial scan showed hyperechogenic lesion (11.5 x 10.5 mm) in the anterior proximal horn associated with a blood clot and the hyperechogenicity of the ventricular wall due a chemical ventriculitis (Figures 1B-1C). The median sagittal scan showed a hypoechogenic central pseudocyst and some hyperechogenic lesions (Figure 1D).

Based on the fetal US findings, the patient opted for the interruption of pregnancy. Given the tracheoesophageal fistula and the history of two previous cesareans, we decided on an abortive cesarean section (CS) at 23 weeks of gestation under general anesthesia; a diagnostic bronchoscopy subsequently confirmed the presence of the fistula. The follow-up of the patient at six months after CS showed a significant regression of long COVID symptomatology, a complete resolution of tracheoesophageal fistula, and the removal of the stoma with the resumption of the intestinal clinical picture. The postoperative period following the CS was not different from that of “healthy patients”.

**Figure 2:**
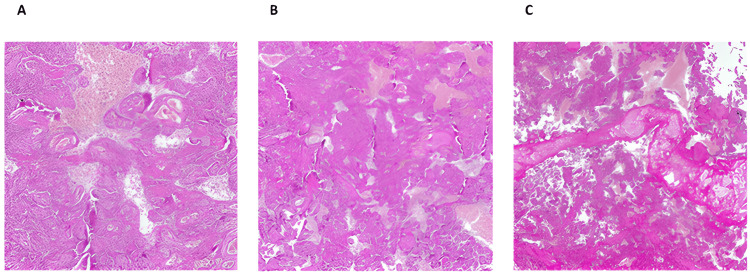
Histological photomicrograph of a chorionic disc from a SARS-CoV-2-positive mother's placenta (A) Note the extensive intervillous fibrin deposition, terminal villous hyperplasia, Chorangiosis, and signs of maternal malperfusion (hematoxylin-eosin, original magnification 4x). (B) Photomicrograph shows extensive and widespread deposition of intervillous fibrin, with chronic histiocytic intervillositis. Note the almost complete obliteration of the maternal/fetal space with a significant reduction in exchanges at the interface (hematoxylin-eosin, original magnification 10x). (C) Histological detail of the previous photomicrograph shows extensive intervillous fibrin deposition, important chronic histiocytic intervillositis, and foci of intravasal thrombosis (hematoxylin-eosin, original magnification 20x) SARS-CoV-2: severe acute respiratory syndrome coronavirus 2

The pathological aspects of the placenta and fetal autopsy after the abortive CS showed that the female fetus, with a weight of 590 g, corresponded to 22 weeks of pregnancy. Macroscopical examination showed a severe intraventricular and parenchymal hemorrhage (grade IV) with evidence of ischemic damage to the encephalus, adrenal glands, and heart. A fair amount of citrine liquid was removed from the chest at its section. The heart was left-located as usual with an average shape and normal atrioventricular and ventricular-arterial connections, even though it appeared ischemic. The diaphragm appeared normal, with internal organs having a regular shape and location. The placenta weighed 250 g and measured 10 x 10 x 40 cm with a central insertion of the umbilical cord, in line with the gestational age. The funicle appeared regular. There was a discrete intervillous deposition of fibrin and space characterized by macrophagic and granulocytic infiltration; intervillous space with deposition of fibrin and high activity of histiocytic cells was observed, with some hotspots of necrotic deciduitis. This morphological appearance was consistent with COVID-19 infection and probable fetal oxygenation compromise related to deciduitis (Figures 2A-2C).

**Figure 3:**
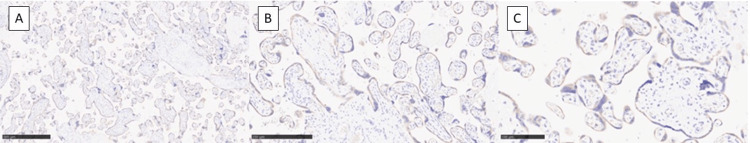
Immunostaining for anti-SARS-CoV-2 S1 glycoprotein (A) Immunostaining showing chorial disc with brown positivity at the level of syncytiotrophoblast cells (immunostaining for SARS-CoV-2, original magnification 4x). (B) Details of the previous image: note the strong positivity for S1 glycoprotein of SARS-CoV-2 at the level of syncytiotrophoblast (immunostaining for SARS-CoV-2, original magnification 10x). (C) Another picture signifying the positivity for SARS-CoV-2 (immunostaining for SARS-CoV-2, original magnification 20x) SARS-CoV-2: severe acute respiratory syndrome coronavirus 2

Moreover, we performed immunostaining with anti-SARS-CoV-2 S1 glycoprotein monoclonal antibody (Thermofisher, clone HL6) on the placenta and fetal encephalon to see if there might have been a transmission. Immunostaining for SARS-CoV-2 was strongly positive at the level of the placental chorionic disc, particularly in the cells constituting the syncytiotrophoblast and in maternal leucocytes (Figures 3A-3C).

On the other hand, immunoreaction was completely negative in the fetal brain tissue. In addition, we tested the brain with the monoclonal recombinant anti-angiotensin-converting enzyme 2 (ACE2) antibody [EPR24705-45] (ab272500), to investigate if there was immunoexpression of ACE2 in the brain. The reaction was completely negative. Furthermore, we performed immunohistochemical investigations on the fetus's heart, lungs, and bowel, which showed negative results. Also, a peripheral blood sampling of the fetus was negative for anti-N and/or S antibodies to SARS-CoV-2.

## Discussion

In the early months of the COVID-19 pandemic, the research predominantly focused on respiratory symptomatology and data from autopsy findings of patients who died from COVID-19; however, as time went on, researchers have tried to investigate the potential impact that SARS-CoV-2 infection could have on pregnant women. This study discusses a rare case of fetal intraventricular hemorrhage following critical maternal COVID-19 infection during the early second trimester of pregnancy accompanied by negative findings related to other infective agents.

We reported the most severe COVID-19 presentation among the entire population in our previous monocentric and cross-sectional study involving 287 patients conducted at the COVID-19 Division of the Obstetrical and Gynecological Unit and Intensive Care of Policlinico di Bari, in Bari, Italy, between September 2020 and April 2022 [9]. Notably, the patient contracted COVID-19 during the second wave of the pandemic, dominated by Alpha VOC (Variant of Concern), which was characterized by a higher risk ratio (odds ratio: 0.03) for ICU admission in comparison with the previous wild variant [11-12]. A recent population-based prospective cohort study involving 315 patients compared the effects of the infection from the wild type on maternal and perinatal outcomes with those of the Alpha VOC; during the Alpha wave, the use of ventilatory support and/or ICU hospitalization significantly rose [11]. Indeed, the ICU step-up prevalence of pregnant women increased from 16.5% in the first wave (wild) to 21.1% during the second wave (Alpha- and Beta-dominant), peaking at 62.4% during the dominance of the Delta VOC in the third wave (p<0.001) [12].

In our case, the ventriculomegaly was probably related to obstruction of the ventricular outlets by the clots debris. Fetal subependymal/intraventricular hemorrhage occurs in one in 10.000 pregnancies with a higher prevalence in premature infants and ranges in severity from germinal matrix hemorrhage (grade 1) to extension of the hemorrhage to the parenchyma as a hyperechoic area (grade 4). In our case, the differential diagnosis from other types of ventriculomegaly was the detection of clots inside the enlarged ventricles and the typical appearance of the ependymal walls with hyperechoic and irregular margins due to chemical ventriculitis caused by the blood. Although visualization of blood clots in the ventriculomegaly is used for differentiating hemorrhage from other causes of ventriculomegaly [13], we needed to differentiate this US hemorrhagic aspect from infective US aspects. Moreover, the screening for thrombophilia was negative. Therefore, we tested TORCH pathogens antibodies (avidity test as well), which returned negative results. Moreover, DNA PCR tests on blood and urine samples for CMV, HSV1, HSV2, HIV, and parvovirus B19 were all negative.

Four different studies related to fetal intraventricular hemorrhage in concomitance with SARS-CoV-2 infection are present in the current literature [14-17]. A recent case report described an exceptional vertical transmission of SARS-CoV-2 during the second trimester (21st week) complicated by severe fetal growth restriction, ultrasonographic features of leukomalacia in hypoxic-ischemic brain injuries with periventricular cyst until the onset, as in our case, of intraventricular hemorrhage at the 25th weeks [14]. Similarly, a case with fetal US evidence of hydrocephalus and porencephalic cysts with a neonatal autoptic demonstration of intraventricular hemorrhage was reported after severe maternal COVID-19 infection during the second trimester (25+5) [15].

Even when they are not pathognomonic, there are some frequent histological patterns with regard to COVID-19-infected placentas: acute/chronic inflammation (34.7%), fetal vascular malperfusion (FVM) (9.2%), maternal vascular malperfusion (MVM) (37.8%), and others features (16.3 %), such as villous edema (6.3%) [4]. A large double-blinded case-control study (71:142) recently conducted at our center investigated the histopathological features of the placenta in pregnant women affected by COVID-19 infection [4]. Notably, the FVM was significantly higher in cases than in healthy controls (21.1% vs. 4.2%, p<0.001), whereas the MVM showed only a non-significant trend in cases vs. controls (54.3% vs. 43.7%, p=0.19) [3]. Furthermore, in cases of COVID-19 infection, the placenta had a significantly higher rate of decidual arteriopathy (40.9% vs. 1.4%, p<0.0001), decidual inflammation (32.4% vs. 0.7%, p<0.0001), perivillous fibrin deposition (36.6% vs. 3.5%, p<0.0001), and fetal vessel thrombi (22.5% vs. 0.7%, p<0.0001) [4]. The anti-SARS-CoV-2 spike-S1 glycoprotein antibodies were prevalent in the cytoplasm of the villi trophoblasts (65% diffuse, 35% focal positivity). Moreover, they were also found in 25% of cases in the endothelium of the villi capillaries in case of thrombosis, maternal decidual cells, as well as in the intervillous histiocytes. Interestingly, electron microscopy evidenced viral structures (spherical formations with a diameter of 100-130 nm surrounded by peripheral electron-dense spicules) in syncytial cells in association with gross vacuolization of the cytoplasm.

Most of the histological alterations (FVM, perivillous fibrin deposition, and fetal vessel thrombi) are maternal immune-mediated phenomena, whereas MVM can be considered an adaptational modification [4,8,16]. The onset of severe and extensive placentitis is a rare COVID-19-related scenario and affects more than 75% of the maternal intervillous space leading to placental insufficiency, fetal asphyxia, and stillbirth without fetal infection [16-17]. As in our case, this is significantly associated with the presence of the Alpha variant, and recently also with the Delta variant [18]. Even in cases of possible placental infection, the fetus has many defense tools: the absence of intercellular gap junction within the syncytiotrophoblast layer, the presence of trophoblastic basement membranes, and the innate immune system [3-4]. In other words, maternal infection can cause viremia leading to placental infection and, in exceptional cases, vertical transmission.

Placental infarction is related to early COVID-19 infection in neonates (<24 hours), but not to the severity of maternal symptoms [2]. The exceptional maternal viremia during COVID-19 infection seems to protect the fetus [10], probably together with the shift of the maternal immune response from T1 to T2 [18,19]. However, the eventual presence of anti-COVID-19 IgM in the cord serum can be associated with placental-barrier dysfunction [10]. Prelabour rupture of the membrane may allow the intrauterine infection of the fetus with a massive chronic intervillositis, especially if it is premature, but the mechanism behind it remains unclear [2]. In fact, only the presence of severe or critical COVID symptoms correlates with a higher rate of preterm births (p=0.03) and respiratory distress syndrome among newborns even without any positivity for COVID-19 infection [17].

In the study previously conducted at our center by Resta et al., even in the case of placental positivity for COVID-19, the neonates were all negative for COVID-19 on nasopharyngeal swabs and the Apgar scores at one minute and five minutes were not significantly different (p=0.83) between cases and controls [4]. A multicentric Italian study has reported that the PCR positivity for COVID-19 on neonatal nasopharyngeal swabs was extremely rare with a rate of 0.0-6.0 %, which is lower than in a recent review that reported a rate of 92% negative neonates from positive mothers. Notably, the second swab performed after one month after birth was mainly negative (96-99%) even in cases with breastfeeding [17], encouraging the rooming-in practice. No positivity to COVID-19 has been found on vaginal swabs or in human milk [9,19].

Regarding immunohistochemical reactions, of great interest is both the demonstration of strong positivity for S1 spike glycoprotein of SARS-CoV-2 at the level of the placental disc and maternal leucocytes and the negativity at the level of fetal chorial villi and fetal encephalon. This feature is different from the one reported by Massimo et al. [16] that, in our cases, described the presence of SARS-CoV-2, particularly, at the level of the choroid plexus. Moreover, ACE2 negativity at the encephalic level may be of some importance in relation to possible speculations of SARS-CoV-2 transmission in this district and beyond [20-21].

The membrane-bound ACE2 acts as a SARS-CoV-2 receptor, whereas the transmembrane serine protease 2 (TMPRSS2) behaves as an activating cofactor for viral cell entry via endocytosis, thanks to the cleavage of the viral spike protein [19-22]. ACE2 is located in most organs such as the heart, lungs, kidneys, vessels, brain, and others, including the placenta, mainly governing vascular and heart functions [22]. ACE2 is located weakly only at the stromal side of the syncytiotrophoblast of the trophoblast villous, whereas it is quite indetectable on the maternal endothelial side that hosts few TMPRSS2 receptors [10], partially explaining the extremely rare vertical transmission [16,23]. The main biological role of ACE is to convert angiotensin I (Ang I) to angiotensin II (Ang II), which binds AT1 receptors [24]. At the same time, ACE2 produces angiotensin 1-7 (Ang 1-7) that inhibits AT II via the activation of the anti-inflammatory MAS receptors [24]. Ang 1-7 determines vasodilating, anti-inflammatory, anti-fibrotic, and anti-thrombotic effects [24]. From a biological viewpoint, the presence of comorbidities (diabetes, preeclampsia, cardiovascular disease, and hypertension) or older age is associated with a downregulation of ACE2, leading to an easier disruption of the ACE-Ang II-AT 1 pathway that generates vasoconstriction, inflammation, fibrosis, edema, and lung damage due to reduced production of Ang (1-7) by ACE2 that inhibits AT II [24].

Notably, the placental expression of ACE2 and TMPRSS2 decreases during pregnancy, suggesting a differential risk of viral infection based on gestational age [25], mainly depending on a higher activity of the disintegrin and metalloprotease domain 17 (ADAM17) or tumor necrosis factor-A (TNF-α) converting enzyme since the early II trimester [26]. As in our case involving critical maternal α infection with fetal intraventricular hemorrhage, the onset of severe placentitis is possible given the high-intermediate rates of placental ACE2 and spike TMPRSS2 during the early II trimester [26]. Even though we have not directly demonstrated the evidence of vertical transmission, we have found a typical placental COVID-19-related scenario with indirect signs of infection that led to placental and fetal hypoxia.

The main issue of COVID-19 infection during pregnancy is due to maternal respiratory symptoms with the consequent hypoxia, viremia with proinflammatory cytokines storm including interferon-G (IFN-G), interleukin-2 (IL-2), IL-6, IL-7, IL-10, and TNF-α, endothelial dysfunction, placental hypoperfusion/ischemia/inflammation leading to a higher rate of maternal hospitalization at ICU, worse perinatal outcomes, and theoretically long-term neurodevelopmental sequelae (i.e., autism, psychosis, and neurosensorial deficits) after the dramatical onset of the fetal inflammatory response (FIRS) mainly due to the rising level of IL-6 [3].

A recent report has described the case of a dichorionic twin pregnancy complicated by COVID-19 infection about two weeks before SVB at 30 weeks + four days of GA [8]. The first neonate was born alive and viable, whereas the second went through severe intrapartum distress that ended with death after a few minutes. Both the placentas showed pathological aspects, especially along the intervillar space with deposition of fibrin and high activity of histiocytic cells, which can compromise fetal oxygenation, leading to a fatal outcome only in the second twin, probably due to the longer expulsive phase and the ischemia sustained by a higher number of total contractions. Thus, we can assume that the effect of maternal COVID-19 infection on the fetal brain is indirect, due to the maternal inflammatory response and chorioamnionitis leading to intraventricular hemorrhage, as in our case, or even cortex/hippocampus alterations leading to schizophrenia, autism, and other behavioral disorders linked to pre-pulse deficits in the offspring [3,25].

With regard to this field, a paper by Faure-Bardon et al. [27] discusses the presence and expression of ACE2 protein in fetal and placental tissues during gestation and how this might influence the vertical transmission of SARS-CoV-2 virus from mother to fetus during pregnancy. Starting from the fact that pregnant women can be infected with the SARS-CoV-2 virus, the article aims to evaluate the protein expression of ACE2 in the placenta and fetal organs during pregnancy, both in SARS-CoV-2 uninfected cases and in a placenta of an infected woman. Therefore, an immunohistochemistry analysis of ACE2 is performed on fetal and placental tissue samples from a biobank licensed by the National Biotechnology Agency, including fetal and placental organ samples from SARS-CoV-2 uninfected pregnancies. Two placentas were also analyzed, one from a woman with a miscarriage and the other from a woman who was RT-PCR-positive for SARS-CoV-2. The authors reported that the protein expression of ACE2 was detected in fetal tissues of the testes, kidneys, intestines, and placenta, but not in the brain. In pediatric control tissue samples, a similar expression was found. In samples from uninfected placentae, ACE2 was detected in the syncytiotrophoblast and cytotrophoblast, but not in the vascular endothelium.

The article argues that SARS-CoV-2 could cross the placenta at any stage of pregnancy, either through blood transmission or through the maternal-fetal interface. However, it seems unlikely that the virus could cause direct neurological damage because ACE2 is not expressed in the brain. Expression of ACE2 in fetal kidneys could influence amniotic fluid production and renal development. The article concludes that the findings provide information on the possible vertical transmission pathway of SARS-CoV-2 and its potential implications.

Interestingly, a very recent study by Cicco et al. [28] attempted to shed light on the mechanisms underlying the alteration of coagulation parameters (so-called Virchow's triad) present in the vascular bed of patients with SARS-CoV-2. In particular, starting from a histological and immunophenotypic study, the authors investigated, through immunohistochemical investigations, the expression of some molecules that might be responsible for some of the most typical manifestations, including pulmonary thromboembolism. In doing so, by correlating clinical (anesthesiological and resuscitation) parameters, it was understood that in patients with higher expression and release of high mobility group box 1 (HMGB1) protein, there was an increased ease in terms of determining hypercoagulative states, which are then responsible for the majority of vascular problems in COVID-19 patients. This paper is, in our opinion, also of importance to our field as it could provide an explanation as to why cytokine/inflammatory storm can lead to a hyperinflammatory state and thus sequelae in the fetus/infant.

Based on the above findings and observations, we believe that a possible future direction in research on SARS-CoV-2 infection in pregnant women could take into account the study of these molecules in the placental vascular bed of COVID-19-positive and (especially) symptomatic women, thereby investigating whether immune dysregulation of HMGB1 could really lead to the coagulopathic effects observed in this cohort of patients, as well as in the other groups of SARS-CoV-2-infected subjects.

## Conclusions

In this report, a typical placental COVID-19-related scenario showed indirect signs of infection leading to placental and fetal hypoxia associated with a critical case of early second-trimester COVID-19 (VOC α) maternal outcome and fetal intraventricular hemorrhage, even without a direct demonstration of vertical transmission. Therefore, among the possible effects of maternal COVID-19 infection, placental damage with deciduitis and secondary fetal oxygenation impairment and proinflammatory activation, a fetal massive intraventricular hemorrhage should also be considered, warranting attention to US signs of ventriculomegaly, hyperechoic and irregular ependymal walls, and clots inside the ventricles.
